# Manipulation of arterial stiffness, wave reflections, and retrograde shear rate in the femoral artery using lower limb external compression

**DOI:** 10.1002/phy2.22

**Published:** 2013-07-08

**Authors:** Kevin S Heffernan, Wesley K Lefferts, Ari G Kasprowicz, Brendan J Tarzia, Dick H Thijssen, Tom D Brutsaert

**Affiliations:** 1 Department of Exercise Science, Syracuse UniversitySyracuse, New York; 2 Department of Physiology, Radboud University Nijmegen Medical CentreNijmegen, The Netherlands; 3 Liverpool John Moores University, Research Institute for Sport and Exercise SciencesLiverpool, U.K

**Keywords:** Arterial stiffness, hemodynamics, intima-media thickness, wave reflections

## Abstract

Exposure of the arterial wall to retrograde shear acutely leads to endothelial dysfunction and chronically contributes to a proatherogenic vascular phenotype. Arterial stiffness and increased pressure from wave reflections are known arbiters of blood flow in the systemic circulation and each related to atherosclerosis. Using distal external compression of the calf to increase upstream retrograde shear in the superficial femoral artery (SFA), we examined the hypothesis that changes in retrograde shear are correlated with changes in SFA stiffness and pressure from wave reflections. For this purpose, a pneumatic cuff was applied to the calf and inflated to 0, 35, and 70 mmHg (5 min compression, randomized order, separated by 5 min) in 16 healthy young men (23 ± 1 years of age). Doppler ultrasound and wave intensity analysis was used to measure SFA retrograde shear rate, reflected pressure wave intensity (negative area [NA]), elastic modulus (Ep), and a single-point pulse wave velocity (PWV) during acute cuff inflation. Cuff inflation resulted in stepwise increases in retrograde shear rate (*P* < 0.05 for main effect). There were also significant cuff pressure-dependent increases in NA, Ep, and PWV across conditions (*P* < 0.05 for main effects). Change in NA, but not Ep or PWV, was associated with change in retrograde shear rate across conditions (*P* < 0.05). In conclusion, external compression of the calf increases retrograde shear, arterial stiffness, and pressure from wave reflection in the upstream SFA in a dose-dependent manner. Wave reflection intensity, but not arterial stiffness, is correlated with changes in peripheral retrograde shear with this hemodynamic manipulation.

## Introduction

The peripheral blood flow waveform in human conduit arteries is triphasic, exhibiting characteristic antegrade (forward) flow during systole and a combination of retrograde (backward) and secondary antegrade flow during early and late diastole, respectively (Mahler et al. 1977; Marquis et al. 1984; Hashimoto and Ito 2010). Whereas antegrade shear is associated with an antiatherosclerotic phenotype, increased retrograde shear is associated with endothelial dysfunction and a proatherogenic phenotype (Gnasso et al. 1996; Kornet et al. 1999; Thijssen et al. 2009; Duivenvoorden et al. 2010; Chiu and Chien 2011). Although highly investigated of late (Padilla et al. 2010, 2011; Young et al. 2010; Casey et al. 2012), the vascular and hemodynamic correlates of retrograde shear in peripheral conduit vessels remain poorly understood (Halliwill and Minson 2010).

Pressure from wave reflections is a potentially important hemodynamic factor influencing the contour of the flow waveform. With each cardiac contraction, a pressure wave is produced that traverses the systemic arterial tree. Bifurcations and changes in peripheral vascular/arteriolar tone result in reflection of the pressure wave. It is generally acknowledged that pressure from wave reflections augment incident wave pressure but attenuate antegrade flow (Westerhof et al. 1972; O'Rourke and Avolio 1980; Westerhof and O'Rourke 1995). Whether pressure from wave reflections is associated with retrograde shear in vivo has not been as extensively studied. Arterial stiffness may also affect the blood flow profile in the systemic circulation. Transmission of blood into an elastic conduit artery results in radial expansion and recoil of the vessel, converting pulsatile flow into laminar flow. With an increase in stiffness, this buffering capacity is lost resulting in increased flow pulsatility and altered shear patterns (Nichols et al. 2011). Examination of arterial stiffness and wave reflections as prominent correlates of retrograde shear in the peripheral circulation will provide novel insight into the hemodynamic genesis of pro- and antiatherosclerotic shear profiles in vivo.

Inflation of a pneumatic compression cuff to subdiastolic pressures has been demonstrated to effectively increase retrograde shear in a dose-dependent manner (Thijssen et al. 2009; Tinken et al. 2009; Carter et al. 2013; Johnson et al. 2013). External compression has also been used to create physical wave reflection sites and manipulate pressure from wave reflections and arterial stiffness in upstream vessels (Latham et al. 1985; Heffernan et al. 2007). Exploitation of this technique may thus provide a novel model to explore the in vivo relation between localized changes in arterial stiffness and pressure from wave reflections with retrograde shear (Klanchar et al. 1990; Wang and Tarbell 1995). The purpose of this study was to explore the association of superficial femoral artery (SFA) stiffness and pressure from wave reflections with SFA retrograde shear rate during manipulation of regional hemodynamics with distal cuff compression. We hypothesized that external cuff compression would increase SFA retrograde shear in a stepwise fashion and this would be accompanied by dose-dependent increases in SFA stiffness and pressure from wave reflections.

## Methods

### Participants

Sixteen healthy men from the University community participated in this study (23 ± 1 years, body mass index 26 ± 1 kg/m^2^). Exclusion criteria included self-reported (from a health history questionnaire) smoking, hypertension, diabetes mellitus, hyperlipidemia, pulmonary disease, renal disease, neurological disease, and peripheral artery disease. Participants were not taking medications of any kind. This study was approved by the Institutional Review Board of Syracuse University, and all subjects provided written informed consent before study initiation.

### Experimental protocol

All testing was conducted in a quiet, dimly lit, temperature-controlled laboratory. Participants were instructed to fast for ≥3 h and avoid vigorous exercise and consuming caffeine or alcohol on the day of testing. Upon arrival, participants rested for 10 min in a supine position. The treatment leg was supported by two foam blocks under the upper leg and ankle. The SFA was chosen for imaging given its clinical significance as an atherosclerosis-prone vessel and its location distal to the confounding influence of prominent upstream reflection sites and sources of turbulent flow (aortoiliac, iliofemoral bifurcations) (Greenwald et al. 1990; Pythoud et al. 1996). A pneumatic compression cuff was tightly secured around the calf at the area of greatest circumference. The calf was selected for the following reasons: (1) to remove the potentially confounding influence of regional femoral compression on local vascular properties (Heffernan et al. 2007); and (2) the calf region is a fundamental source of discrete reflection sites, therefore exogenous compression would be expected to amplify endogenous reflection (O'Rourke and Taylor 1966). The cuff was attached to a rapid cuff inflation system (Hokanson, Bellevue, WA). Participants underwent the following three cuff compression conditions in a randomized order: 0, 35, and 70 mmHg. These pressures were selected based on recent studies using a similar compression schema in the brachial artery to manipulate shear profiles (Thijssen et al. 2009). Pilot investigation in our lab (*n* = 3) demonstrated that a pressure of 70 mmHg was sufficient to alter the SFA flow profile. Each intervention was approximately 5 min in duration and was separated by 5 min of rest between conditions. The treatment leg (left vs. right) was randomized. SFA measurements of diameter, flow velocity, and wave intensity were taken ∼2 min after the start of cuff inflation to ensure steady-state conditions, as acute venous emptying from external compression may alter vascular function (Tschakovsky and Hughson 2000). This was confirmed using a qualitative substudy described below.

### Vascular measurements

Images of the SFA were obtained using Doppler ultrasound (ProSound α7, Aloka, Tokyo, Japan) attached to a 5.0–13.0 MHz linear array probe during cuff compression conditions. The artery was imaged ∼8–10 cm distal to the bifurcation of the common femoral artery. Wave intensity analysis (WIA) combined with eTracking was used to derive measures of forward and reflected wave intensity and arterial stiffness. This method has been described in detail previously (Harada et al. 2002; Niki et al. 2002). Briefly, this technique simultaneously measures SFA distension waveforms (analogous to the pressure waveform) and flow waveforms. The distance from the near-wall to far-wall lumen–intima interface was continuously traced using eTracking software. The echo-tracking system measures diameter changes within 1/16th of an ultrasound wavelength (0.013 mm) (Ohte et al. 2003) creating a distension waveform almost identical to pressure waveforms (Van Bortel et al. 2001). WIA distension waveforms were calibrated using brachial blood pressure (from simultaneous oscillometric recordings) during each condition. Flow waveforms were measured using range-gated color Doppler signals averaged along the Doppler beam. An insonation angle ≤60° was maintained for all measures. Sample volume was adjusted to encompass the entire vessel. At least eight waveforms were ensemble averaged to gain a representative average waveform. Wave intensity was calculated using time derivatives of blood pressure (*P*) and velocity (*U*), where wave intensity = (d*P*/d*t* × d*U*/d*t*); thus, the area under the d*P*/d*t* × d*U*/d*t* curve represents the energy transfer of the wave (Sugawara et al. 2009). WIA states that if these wavefronts carry a positive rate of pressure change, they are referred to as compression waves. Conversely, if the wavefront carries a negative rate of pressure change, they are referred to as expansion waves. It should be noted that “expansion” in this setting is an expression from fluid dynamics theory referring to “decreasing pressure” and not to be confused with “dilitation (Sugawara et al. 2009).” (1) W_1_ represents a forward compression wave produced during early systole that accelerates flow and increases pressure; (2) W_2_ represents a forward expansion wave that decelerates flow and reduces pressure; (3) the negative area (NA) between W_1_ and W_2_ is a backward traveling compression wave due to the sum of waves reflected from the periphery (wave reflection intensity) that decelerates flow but increases pressure. The time interval between the R-wave of the ECG and W_1_ is analogous to the pre-ejection period (Niki et al. 2002) and was used as a crude proxy of peripheral sympathetic activation (Schachinger et al. 2001). Results obtained from WIA are highly reproducible (Liu et al. 2011a,b) and have been shown to be analogous to results obtained using traditional linear wave separation with impedance analysis for the determination of forward and backward traveling waves (Hughes and Parker 2009).

Arterial stiffness measures included stiffness index (β), Peterson's pressure-strain elastic modulus (Ep), and a single-point pulse wave velocity (PWV). All variables were automatically calculated by the echo-tracking subsystem using the formulas listed below:


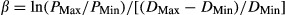



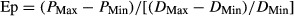



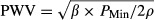


where *P* and *D* correspond to pressure and diameter, respectively, and Max and Min refer to maximum (systolic) and minimum (diastolic) values during the cardiac cycle. Blood density, ρ, is assumed constant and equals 1050 kg/m^3^. We additionally computed the reflection coefficient (RC) as RC = NA/W_1_ + NA as a proxy of distal vascular tone (Liu et al. 2011a).

Systolic antegrade, diastolic retrograde, diastolic antegrade, and mean blood velocities (*V*m) were measured using Doppler ultrasound as described above and calculated as follows: *V*_m_ = ∫ *V*(*t*) d*t*/FT, where ∫ *V*(*t*) d*t* is the velocity–time integral of the velocity waveform and FT is flow time. Shear rate was calculated as 4 × (*V*m/Diameter). SFA intima-media thickness (IMT) was assessed using a longitudinal view of the artery with both near-wall and far-wall lumen–IMT boundaries clearly visible. Once a satisfactory image was obtained, wall thickness was measured separately during systole and again during diastole (determined from simultaneous ECG gating) across a continuous 5-mm region of interest via semiautomated digital calipers. The distance from the lumen–intima interface to the media–adventitia interface was taken as the IMT.

### Systemic hemodynamic measures

Beat-to-beat systolic and diastolic blood pressure (SBP and DBP, respectively) were monitored continuously using digital photoplethysmography (Finapres Medical Systems, Amsterdam, The Netherlands) to ensure that the intervention did not alter systemic hemodynamics (Guelen et al. 2008). Arterial pressure in the finger was measured via the volume-clamp method, which is based on the development of the dynamic pulsatile unloading of the finger arterial walls (Guelen et al. 2003). Beat-to-beat blood pressure measurements were calibrated to brachial pressures prior to experimental testing. The Modelflow method was used to derive stroke volume and cardiac output (heart rate × stroke volume) and total peripheral resistance (mean arterial pressure/cardiac output) (Bogert and van Lieshout 2005). Brachial blood pressure was further obtained via a validated automated oscillometric cuff (EW3109, Panasonic Electric Works, Secaucus NJ) at baseline and immediately pre–post femoral imaging for each compression condition (Bonso et al. 2010).

### Acute substudy

In order to gain qualitative insight into hemodynamic temporal changes instigated by direct cuff compression, a subset of participants (*n* = 6) reported to the laboratory on a separate day and underwent trials at 0-mmHg and 70-mmHg compression. This pressure was selected as it was hypothesized to cause the most robust changes in vascular and hemodynamic parameters. Due to technical requirements of WIA, instantaneous data could not be generated. Therefore, a combination of Doppler ultrasound and tonometry (Sphygmocor, Atcor Medical, Australia) was used to simultaneously capture flow velocity waveforms and pressure waveforms in the left and right femoral artery, respectively, during dual lower limb compression (Hokanson, Bellevue, WA). Due to the overall smooth contour of the resting femoral pulse waveform and lack of a clear inflection point, calculation of global wave reflection using standard metrics (i.e., augmentation index) was not feasible. Data images are displayed for visual inspection only.

### Statistical analyses

All data are reported as mean ± standard error of the mean and statistical significance was established a priori as *P* < 0.05. A one-way analysis of variance (three conditions; 0, 35, and 70 mmHg) was used to analyze main outcome variables. If a significant main effect was detected, post hoc comparisons were made using the Tukey method. Associations of interest were examined across conditions using Pearson's correlation coefficients. Absolute change scores were computed as (1) 70-mmHg compression values – 0-mmHg compression values and (2) 35-mmHg compression values – 0-mmHg compression values. Change scores across conditions were combined to examine interassociations between vascular and hemodynamic parameters. To explore day-to-day variability in primary vascular and hemodynamic measures, paired samples *t*-tests were used to compare means. Intraclass correlation coefficients were used to gauge reliability. All statistical analyses were carried out using IBM SPSS version 20 (SPSS Inc., Chicago, IL).

## Results

Day-to-day repeatability of hemodynamic measures calculated from a subset of subjects (*n* = 6) on two separate days was fair (intraclass correlation coefficients of 0.65–0.94 for all) and resting values did not differ between testing days (*P* > 0.05). There were no differences in brachial SBP or DBP measured immediately before and after each condition (Table 1). Similarly, there were no differences in SBP, DBP, cardiac output, total peripheral resistance, R-W_1_, or SFA diameters measured across condition (Table 2).

**Table 1:** Brachial oscillometric blood pressure before and after each condition

	0 mmHg		35 mmHg		70 mmHg		
				
	Before	After	Before	After	Before	After	*P*-value
SBP, mmHg	119 ± 1	119 ± 1	118 ± 1	118 ± 1	119 ± 1	120 ± 1	0.94
DBP, mmHg	73 ± 1	73 ± 1	73 ± 1	73 ± 1	74 ± 1	72 ± 1	0.93

**Table 2:** Systemic hemodynamics measured by digital plethysmography during each condition

	0 mmHg	35 mmHg	70 mmHg	*P*-value
SBP, mmHg	126 ± 1	126 ± 1	126 ± 1	0.99
DBP, mmHg	73 ± 1	74 ± 1	73 ± 1	0.80
CO, L min^−1^	6.1 ± 0.3	6.0 ± 0.3	6.1 ± 0.4	0.99
TPR, mmHg L^−1^ min^−1^	1331 ± 68	1344 ± 69	1315 ± 82	0.96

CO, cardiac output; TPR, total peripheral resistance.

### Shear rate

**Figure 1:**
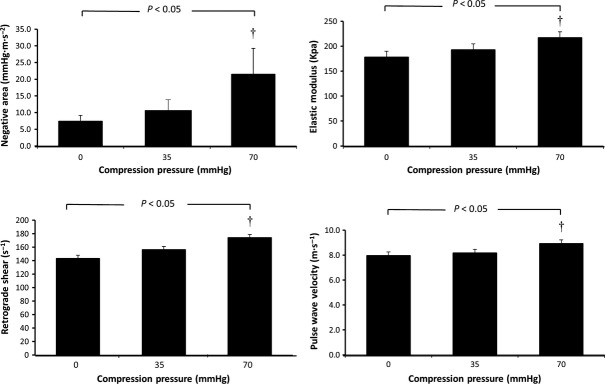
Retrograde shear, arterial stiffness parameters (PWV, pulse wave velocity; Ep, elastic modulus), and wave reflection intensity (NA, negative area) across conditions. ^†^Significantly different than 0 mmHg (*P* < 0.05).

There were significant differences in retrograde shear rate, diastolic antegrade shear rate, and consequently mean shear rate across conditions (Table 3 and Fig. 1, *P* < 0.05). Post hoc pair-wise comparisons revealed that retrograde shear was higher during the 70-mmHg compression condition compared with the 0-mmHg compression condition (Fig. 1, *P* < 0.05), whereas diastolic antegrade and mean shear were lower during the 70-mmHg compression condition compared to the 0-mmHg compression condition (Table 3, *P* < 0.05).

**Table 3:** SFA vascular and hemodynamic parameters across conditions

	0 mmHg	35 mmHg	70 mmHg	*P*-value
Systolic diameter, mm	5.83 ± 0.13	5.86 ± 0.12	5.84 ± 0.13	0.98
Diastolic diameter, mm	6.04 ± 0.11	6.00 ± 0.14	5.93 ± 0.13	0.94
Systolic IMT, mm	0.34 ± 0.02	0.35 ± 0.01	0.34 ± 0.02	0.79
Diastolic IMT, mm	0.34 ± 0.02	0.35 ± 0.01	0.34 ± 0.02	0.94
Systolic antegrade shear rate, sec^−1^	321 ± 23	314 ± 25	340 ± 13	0.63
Diastolic antegrade shear rate, sec^−1^	76 ± 4.9	62 ± 5^1^	16 ± 6^1^,^2^	0.001
Mean shear rate, sec^−1^	102 ± 9	69 ± 7^1^	37 ± 8^1^,^2^	0.001
β-stiffness, aU	13.8 ± 0.9	15.6 ± 1.1	17.2 ± 1.1	0.056
W_1_, mmHg m sec^−3^	7.7 ± 0.5	8.4 ± 0.9	8.8 ± 0.7	0.32
W_2_, mmHg m sec^−3^	1.7 ± 0.2	1.9 ± 0.2	2.3 ± 0.2^1^,^2^	0.031
Reflection coefficient, aU	0.36 ± 0.06	0.46 ± 0.05	0.50 ± 0.07^1^	0.047
R-W_1_, msec	242 ± 6	241 ± 6	245 ± 5	0.86
Heart rate, bpm	61 ± 2	62 ± 2	61 ± 2	0.92

1 Significantly different than 0 mmHg (*P* < 0.05).

2 Significantly different than 35 mmHg (*P* < 0.05).

### Arterial stiffness and wave reflections

There were significant differences in PWV, Ep, W_2_, NA, and RC across conditions (Table 3 and Fig. 1, *P* < 0.05). Post hoc pair-wise comparisons revealed significantly higher values for these parameters during the 70-mmHg compression condition compared with the 0-mmHg compression condition (*P* < 0.05).

### Correlations of shear patterns and vascular hemodynamics

As seen in Table 4, across conditions retrograde shear was associated with NA (*P* < 0.05) and W_2_ (*P* < 0.05). Moreover change in retrograde shear was associated with change in NA (*r* = 0.43, *P* < 0.05) and change in W_2_ (*r* = 0.39, *P* = 0.06). There were no associations between retrograde shear and stiffness parameters (across conditions or change between conditions; all *P* > 0.05). Given colinearity between arterial stiffness measures (all three measures are derived from diameter and blood pressure), PWV was selected as a representative measure of arterial stiffness for correlational display purposes.

**Table 4:** Correlation matrix for hemodynamics and shear components across conditions

*n* = 48	PWV	W_2_	NA	Systolic antegrade shear	Retrograde shear
W_2_	**0.36**^1^				
Negative area	**0.34**^1^	0.13			
Systolic antegrade shear	**0.33**^1^	0.15	0.06		
Retrograde shear	0.18	**0.24**^1^	**0.40**^1^	**0.37**^1^	
Diastolic antegrade shear	**−0.27**^1^	**−0.36**^1^	0.09	0.07	**−0.25**^1^

1 Significant association, *P* < 0.05.

### Compression substudy

**Figure 2:**
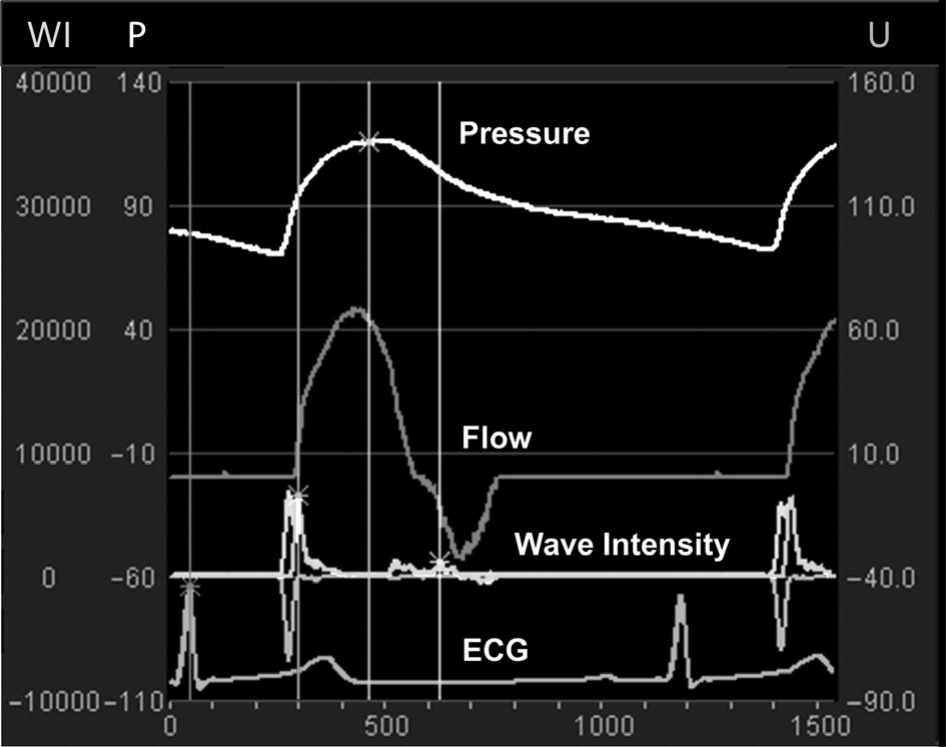
Sample wave intensity analysis during 70-mmHg compression condition. The displayed signals from top to bottom are as follows: the distention waveform (analogous to a pressure waveform), the flow waveform, the wave intensity, and the ECG. WI, wave intensity; P, pressure; U, flow.

**Figure 3:**
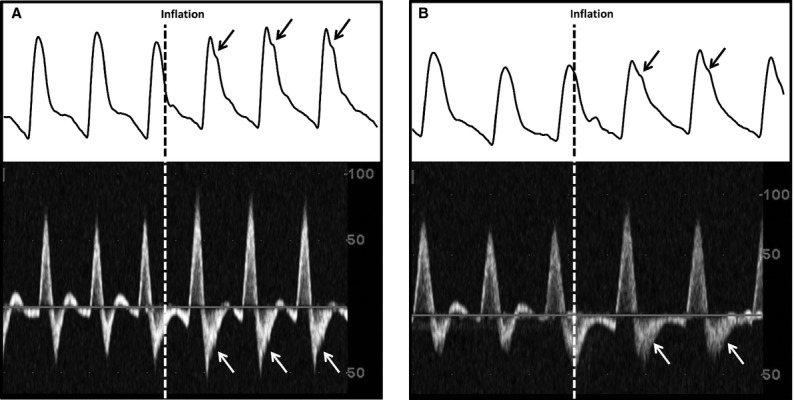
Sample images from two participants showing instantaneous changes in pressure and flow waveforms with external compression to 70 mmHg (denoted “inflation”). Note the immediate appearance of an inflection point on the contour of the pressure wave (white panel, black arrows) signifying increased pressure from wave reflection. Also note the increase in retrograde flow and attenuation of antegrade flow in early and late diastole, respectively (black panel, white arrows).

Figure 2 displays a sample image of WIA during the 70-mmHg compression condition. As can be seen from Figure 3, there was an instantaneous increase in retrograde *V*m (denoted by the arrow) and diminution of diastolic antegrade *V*m with cuff inflation to 70 mmHg. Whereas no clear inflection point could be detected in the femoral pulse waveform during the 0-mmHg compression condition, compression to 70 mmHg produced an instantaneous inflection point on the contour of the pressure wave (also denoted by the arrow) suggesting rapid changes in timing and/or magnitude of pressure from wave reflections. These findings were visually confirmed in all six participants.

## Discussion

Inflation of a blood pressure cuff around the calf successfully induced a stepwise increase in upstream SFA retrograde shear, which confirms previous observations witnessed in the brachial artery (Thijssen et al. 2009; Tinken et al. 2009; Birk et al. 2012). Moreover, we extend this by adding the novel observation that the increase in retrograde shear in the SFA occurs immediately (i.e., within a single cardiac cycle) after cuff inflation. In addition to the stepwise increases in retrograde shear rate, downstream cuff inflation to 35 and 70 mmHg also resulted in concomitant stepwise elevations in wave reflection intensity and arterial stiffness. Changes in pressure from wave reflections also appear to occur rapidly (i.e., within a single cardiac cycle) after cuff inflation. Finally, we demonstrated that changes in SFA retrograde shear are associated with changes in wave reflection intensity but not arterial stiffness.

Current hemodynamic dogma dictates that pressure from wave reflections is a significant determinant of the flow profile in the systemic circulation (O'Rourke and Avolio 1980; Nichols et al. 2011). In vitro simulation studies and mathematical models have proposed that wave reflections not only contribute to flow reversal but also determine the amplitude of vascular wall shear rate (Westerhof et al. 1972; O'Rourke and Avolio 1980; Klanchar et al. 1990; Wang and Tarbell 1995). Retrograde flow is reduced distal from bifurcations (i.e., below wave reflection sites) (Mills et al. 1970; Nichols et al. 2011). In the saphenous, there is no retrograde flow and this has been suggested to be due to distance to downstream reflection sites (Nichols et al. 2011). There is no retrograde flow in the splanchnic branches of the abdominal aorta and this too has been attributed to low peripheral vascular resistance and reflection coefficients in this vascular bed (Nichols et al. 2011). During conditions of reactive hyperemia, there is no retrograde flow in peripheral vessels (Mahler et al. 1977). Similarly, there is reduced magnitude of pressure from wave reflections during reactive hyperemia owing to vasodilation of downstream vessels (Nichols et al. 2011). In these settings, pressure and flow waveforms are virtually identical (Nichols et al. 2011). Our experimental findings using cuff compression to manipulate shear patterns support previous in vitro suggestions that wave reflections are an important correlate of retrograde shear in peripheral conduit vessels in vivo (Mills et al. 1970; Busse et al. 1975).

External compression of the calf also resulted in commensurate increases in upstream SFA stiffness. However, change in SFA stiffness was not a direct correlate of change in retrograde shear per se. SFA PWV was, however, associated with wave reflection intensity and this is in keeping with the notion that arterial stiffness is an important potential moderator of wave reflections (both timing and magnitude). Increases in SFA stiffness were also associated with the near elimination of diastolic antegrade shear, a potential manifestation of diminished arterial reservoir function. With each cardiac contraction, the forward pressure wave that is generated causes radial expansion of the vessel. A portion of the ejected stroke volume is temporarily stored within the vessel wall, and upon cardiac relaxation (i.e., diastole), the vessel recoils and stored blood is expelled back into the systemic circulation. Under resting conditions, retrograde flow serves as the substrate for diastolic antegrade flow and this is facilitated by the arterial reservoir (Hashimoto and Ito 2010). The capacitance or reservoir function of the arterial tree allows for adequate convective acceleration of flow (i.e., “run-off”) to the periphery during diastole ensuring that flow does not fall to zero (Heffernan et al. 2010). Increased arterial stiffness would portend a reduced capacitance function and hence attenuate stored flow discharge into the periphery. This is important as reduced diastolic antegrade shear was associated with increased retrograde shear in this study, and this is evident when viewing Figure 3. Although speculative, arterial stiffness may indirectly affect retrograde shear via its modulatory influence on pressure from wave reflections and diastolic antegrade shear (i.e., the arterial reservoir).

An unanticipated and novel finding was the increase in W_2_ across conditions that were associated with increased retrograde shear and reduced diastolic antegrade shear. W_2_ is a forward traveling expansion wave created by myocardial shortening rate and inertial force of aortic blood flow (momentum) that causes a rapid fall in left ventricle (LV) pressure (Jones et al. 2002; Sugawara et al. 2009). This creates a suction wave that applies a “braking” action to the column of blood from behind and decelerates flow (Jones et al. 2002; Feng and Khir 2008). It has been suggested that the effect of this expansion wave on peripheral flow is modest owing to the physical distance from the source of the suction (i.e., the heart) coupled with wave damping and dissipation (Liu et al. 2011b). However, it is possible that an increase in arterial stiffness and wave speed (less viscous damping and greater dispersion) causes this expansion wave to travel more distally (Jones et al. 2002; Zambanini et al. 2005). Indeed, there was an association between PWV and W_2_ across conditions.

The mechanism responsible for acute increases in SFA stiffness, wave reflection intensity, and retrograde shear with external compression of the calf has not been specifically explored. Given that we noted no change in systolic shear but significant changes in diastolic shear, our findings are in keeping with recent suggestions that this external compression model mimics a quasi-Starling resistor (Halliwill and Minson 2010). According to this theory, external compression reduces regional transmural pressure distending the vessel wall which causes momentary precapillary arteriolar collapse during diastole (when critical closing pressure is attained) and creates a “back pressure” to flow (Shrier and Magder 1995); identified as wave reflection intensity herein. The result is an attenuated diastolic runoff into the periphery and retrograde discharge of stored reservoir flow volume upon vessel recoil. There may also be a reflexive and conducted increase in regional vascular tone aimed at preventing further pressure drops across the vessel wall (Nielsen 1991; Jiang et al. 2011). Indeed, we noted a stepwise increase in the RC suggesting that increased external cuff compression of the lower limb mimics an increase in distal vascular tone (Liu et al. 2011a). In response to low-shear conditions, peripheral conduit vessel tone increases instantaneously in vivo with peak low-shear–mediated constriction occurring approximately 3 min after initial exposure (Levenson et al. 2001). Increased vascular tone from decreased wall shear directly increases arterial stiffness (i.e., low-flow/shear–mediated stiffening) (Joannides et al. 2001). The amplitude/intensity of reflected pressure waves is also directly dependent on the value of peripheral resistance (Westerhof et al. 1972). Changes in arterial stiffness, wave reflection intensity, and retrograde shear may thus have a common origin residing, at least partly, in downstream vascular tone.

The magnitude of change in SFA retrograde shear was somewhat lower than that previously reported in the brachial artery (Thijssen et al. 2009). The reason for this may be related to the amount of tissue compressed, the amount of discrete reflection sites within the particular vascular bed being manipulated, or the physical distance to the effective reflection site (Sugawara et al. 2010). Another possibility resides in potential limb differences between the SFA and brachial artery in the vascular and hemodynamic response to altered transmural pressure (Newcomer et al. 2008). Limb differences have been described regarding the responsiveness to acute altered retrograde shear (Padilla et al. 2009). Future research is needed to explore potential limb differences in vascular and hemodynamic correlates of retrograde shear.

### Limitations

Inflation of a pneumatic compression cuff to alter flow and pressure may not be viewed as cuing a true physiologic response. Our primary purpose was to examine correlates of change in retrograde shear in order to gain deeper insight into underlying mechanism. Other commonly used physiologic perturbations such as cold pressor testing or rhythmic handgrip exercise have not consistently been shown to alter retrograde shear in the upstream brachial artery in young adults (Padilla et al. 2010, 2011). Moreover, increases in heart rate and mean pressure that occur concomitant with cold pressor and exercise are known confounders of change in arterial stiffness, pressure from wave reflections, and retrograde shear (Wilkinson et al. 2000, 2002; Casey et al. 2008; Lydakis et al. 2008; Padilla et al. 2010). We wished to use a method that would enable us to generate a dose–response curve while concomitantly characterizing a fairly isolated regional vascular hemodynamic response devoid of other systemic confounders. Indeed, this perturbation was mild enough not to evoke change in heart rate, stroke volume, pre-ejection period (a proxy of sympathetic activation), systemic vascular resistance, or mean pressure. Another potential limitation is that we did not carry out measures in young women or in older subjects. Therefore, we cannot extrapolate findings to other groups. Finally, this study was not designed to determine if wave reflections directly cause retrograde shear. As seen in Figure 3, changes in retrograde *V*m and pressure wave reflection from limb compression were instantaneous precluding ability to distinguish a temporal order.

On the basis of our findings we propose that retrograde shear in the SFA may be the culmination of: (1) increased resistance “in front” from increased distal vascular tone preventing flow runoff during diastole (Henderson and Johnson 1912); (2) “cessation of push from behind” from an increased expansion wave (suction wave) causing flow deceleration (Henderson and Johnson 1912); and (3) increased wave reflection intensity contributing to flow reversal. In conclusion, experimental manipulation of regional SFA hemodynamics with lower limb external compression results in rapid and dose-dependent increases in upstream SFA retrograde shear and wave reflection intensity. Changes in SFA retrograde shear are associated with changes in wave reflection intensity but not arterial stiffness.
